# Advanced Nanostructured MXene-Based Materials for High Energy Density Lithium–Sulfur Batteries

**DOI:** 10.3390/ijms23116329

**Published:** 2022-06-06

**Authors:** Jingkun Tian, Guangmin Ji, Xue Han, Fei Xing, Qiqian Gao

**Affiliations:** School of Physics and Optoelectronic Engineering, Shandong University of Technology, Zibo 255000, China; wfcltjk@163.com (J.T.); jgm17853318544@163.com (G.J.); hanx216@163.com (X.H.)

**Keywords:** MXene, lithium–sulfur battery, polysulfides, cathode, interlayer

## Abstract

Lithium–sulfur batteries (LSBs) are one of the most promising candidates for next-generation high-energy-density energy storage systems, but their commercialization is hindered by the poor cycling stability due to the insulativity of sulfur and the reaction end products, and the migration of lithium polysulfide. MXenes are a type of emerging two-dimensional material and have shown excellent electrochemical properties in LSBs due to their high conductivity and large specific surface area. Herein, several synthetic strategies developed for MXenes since their discovery are summarized alongside discussion of the excellent properties of MXenes for LSBs. Recent advances in MXene-based materials as cathodes for LSBs as well as interlayers are also reviewed. Finally, the future development strategy and prospect of MXene-based materials in high-energy-density LSBs are put forward.

## 1. Introduction

The global warming and energy crisis caused by the use of fossil fuels has brought us into the era of clean energy revolution. Lithium-ion batteries (LIBs) have developed rapidly since their invention in the 20th century and are widely used in convenient electronic devices and electric vehicles, and dominate the market [1,2]. With the development of science and technology, the growing demand for electronic devices has gradually increased the requirements for battery energy density. The scarcity of transition metal oxide cathodes leads to a high price and limited availability. In addition, the theoretical capacity of a graphite negative electrode is low. Considering the above factors, LIBs are no longer able to meet the future development trend for electronic devices. Therefore, it is necessary to explore high-energy-density electrochemical energy storage systems to respond the market demand [3,4,5]. In this case, lithium–sulfur batteries (LSBs) which have an energy density of 2567 Wh·kg^−1^, about six times that of commercially available LIBs have attracted the attention of researchers. New energy vehicles equipped with LSBs have a longer range on a full charge than the currently commercialized LIBs. Moreover, sulfur has the advantages of low pollution, large reserves and low price. The development of LSBs with high energy density is of great significance [6,7,8,9].

**Figure 1:**
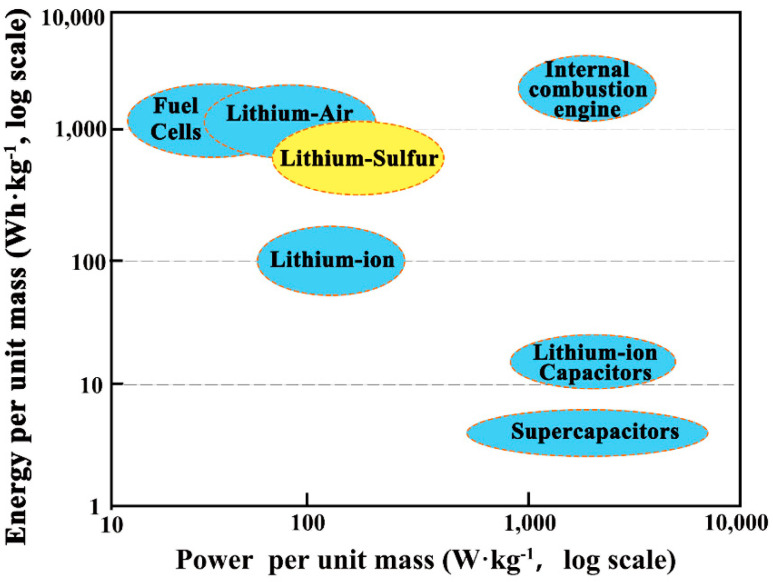
Energy storage Ragone plot.

Energy density and power density are key factors in measuring the potential of an energy storage system, which is often presented in the form of a “Ragone plot” [10]. In Figure 1, we compare the mass energy density and power density of several energy storage systems. It is clear that the internal combustion engine has the highest energy density and power density. Unfortunately, the internal combustion engine converts energy by burning fossil fuels to do work, and the shortage of fossil fuels and the pollution they cause has forced us to turn our attention to other new energy devices. Supercapacitors have a very high power-density, but their energy density is extremely low, so they cannot provide a longer driving range for cars. Fuel cells have the highest energy density but cannot be charged and discharged as quickly as supercapacitors. LIBs are being widely used commercially as energy storage devices with balanced energy density and power density. As a potential energy storage device, LSBs exceed LIBs in both energy density and power density, and can meet the future needs of human beings for energy storage systems.

Unfortunately, multiple reasons such as the serious shuttle effect inside the battery have prevented the large-scale commercial application of LSBs. Firstly, the electronic insulation of sulfur (S_8_) and lithium sulfide (Li_2_S) hinder the redox reaction, and the reduced utilization rate of sulfur leads to poor rate performance [11]. Secondly, the density difference between the final insoluble product, Li_2_S (1.67 g·cm^−3^), formed by discharge and S_8_ (2.03 g·cm^−3^) is large, resulting in nearly 80% fluctuation of electrode volume after multiple charge and discharge processes [9,12]. Furthermore, the repeated stripping of the lithium anode leads to the formation of lithium dendrites, which can easily puncture the separator causing a fatal cell short circuit. Most importantly, the discharge intermediate lithium polysulfide (LiPSs) dissolves in the electrolyte and forms a shuttle effect, which leads to rapid decay of battery capacity and greatly reduces the cycling stability [13,14].

In order to solve these problems, researchers have carried out work in many aspects and adopted various strategies to enhance the cyclic stability of LSBs. For example, materials with high electrical conductivity and chemical adsorption properties were used as sulfur hosts [15,16], functional interlayers were constructed between cathode and separator [17,18,19], optimized electrolytes and built advanced lithium anodes were researched and developed [20,21,22]. Rational design of sulfur host materials was one of the first widely studied strategies. Since Nazar et al. used CMK-3 as a sulfur host material and obtained excellent performance in 2009 [23], combined with the discovery of two-dimensional (2D) material graphene by Geim et al. in 2004, carbon-based materials have been widely used in sulfur cathodes by researchers. Although carbon-based materials make up for the lack of cathode conductivity, they can only be physically encapsulated into LiPSs, which usually leads to poor long-period stability due to their non-polar defects. Then, researchers tried to design polar/nonpolar composites as a sulfur host [24,25,26] or properly use doped carbon materials (such as N, S and B) [27,28,29,30,31,32,33]. For example, Sgroi et al., Rao et al. and Vélez et al. investigated the interaction of heteroatom-doped graphene with LiPSs using the first principle and showed that the introduction of heteroatoms enhanced the adsorption energy between the electrode material and LiPSs [34,35,36]. A large number of reasonable materials have been used repeatedly in sulfur cathode hosts and functional separators. Many researchers have summarized the research progress of carbon-based materials in LSBs. In our previous work, we summarized the research progress of graphene in the field of sulfur cathode in detail, including heteroatom doping of graphene, compounding of graphene with metal compounds and compounding among carbon materials [37]. Zhang et al. summarized the application of carbon materials in more detail from the three fields of LSBs cathode, separator and anode [38]. Although there are so many 2D materials, 2D materials that simultaneously possess high electrical conductivity, high chemisorption, easy production and high yield are still lacking for LSBs.

MXene is a new 2D material composed of transition metal group carbonitrides. It is considered as one of the more competitive candidates for LSBs due to its high electrical conductivity, strong interaction with LiPSs, and easy production. Gogotsi et al. prepared 2D titanium carbide (Ti_3_C_2_) by hydrofluoric acid (HF) etching from Ti_3_AlC_2_ in 2011, marking the advent of Mxene [39]. So far, the family members of Mxene have been expanding continuously, and its etching methods have been successively developed from the previous HF etching to alkali etching [40], electrochemical etching and so on [41]. Due to its excellent properties such as metal conductivity and hydrophilicity, MXene have been widely used by researchers in the fields of electrocatalysis [42], electromagnetism [43] and biomedicine [44]. In the field of energy storage, MXene also shows great potential in energy storage devices such as LIBs [45,46], zinc-ion batteries [47,48], potassium-ion batteries [49,50] and supercapacitors [51,52]. In 2015, Ti_2_C nanosheets were used as a sulfur host material for LSBs for the first time by Nazar et al. The battery still had a specific capacity of 723 mAh·g^−1^ after 600 cycles at 0.5 C, which proved the availability of MXene in LSBs [53]. The review of MXene-based LSBs has also been summarized by many researchers [54,55,56]. For example, Zhao et al. and Xiao et al. summarized in detail the application of MXene materials in cathode, interlayer and anode [57]. In particular, Xiao et al. described in detail a mechanism study of the interaction of MXene terminal functional groups with LiPSs [58]. Up to now, LSBs researchers are still experimenting with various MXene family members in cathodes, anodes and separators. Due to the large number of MXene family members, the mechanism of action of different MXene materials on LSBs varies, so a complete phased summary is particularly critical and is expected to accelerate the development of MXene materials and promote the performance improvement of LSBs.

In this review, we first summarize the different synthesis methods of MXene family materials and analyze the differences in the properties of MXene prepared by different methods. Secondly, the property requirements of LSBs for MXene nanomaterials are also discussed. And most important of all, recent advances in MXene-based materials as cathodes and interlayers for LSBs are also summarized in detail, including pure MXene materials, modified MXene materials and MXene-based composites. Finally, we provide an outlook on how to achieve high cycle life LSBs with respect to the latest advances in MXene-based LSBs.

## 2. Synthesis Strategies and Properties of Nanostructured MXenes

It has been more than a decade since the first Ti_3_C_2_T_x_ (T_x_ represents surface terminations) MXenes were invented. During this time, various methods have been developed for the synthesis of MXenes and many new members of the MXene family have been created. MXene has also been widely used in LSBs due to its superior properties. In this section, we focus on summarizing the recent advances in the synthesis methods of MXenes and theoretical advances in their performance for LSBs applications.

### 2.1. Synthesis Strategies

**Figure 2:**
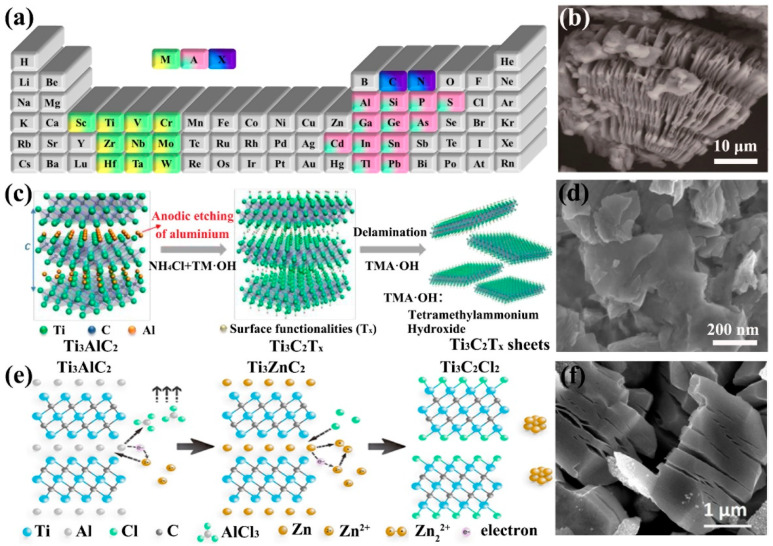
(**a**) Elements represented by MAX respectively. (**b**) SEM image of Ti_3_C_2_ synthesized by HF etching [39]. Copyright 2011, John Wiley and Sons. Synthesis of Ti_3_C_2_T_X_ by electrochemical etching (**c**) and its SEM image (**d**) [72]. Copyright 2018, John Wiley and Sons. Synthesis of MXenes by Lewis acid molten salt etching (**e**) and its SEM image (**f**) [74]. Copyright 2020, American Chemical Society.

MXenes are a general term for transition metal carbides or nitrides, which are prepared by etching the A element in the MAX phase. The MAX phase is called ternary layered compounds, where M represents the transition metal element, A represents an element of the main groups 13 and 14, and X represents the C element or an N element (Figure 2a) [59,60]. Since the binding force between M and X (covalent bond and ionic bond) is greater than that between M and A (metal bond), the A layer is easily etched away, and the rest is MXene [61]. In 2011, Ti_3_C_2_ MXene was first introduced [39]. Since then, HF has become the primary etchant for MAX. However, because of the different bonding strengths between different M elements and different A elements, it is necessary to change the etching conditions to control the synthesis of MXene, including the concentration of HF, reaction time and ambient temperature. In this way, many MXenes are etched, such as: Ti_2_AlC, Nb_4_AlC_3_, Ta_4_AlC_3_, V_2_AlC, etc [62,63,64].

After etching by HF, hydroxyl and fluoride ion reactive groups exist on the surface of transition metal elements in MXene. These end groups play different roles in MXene, resulting in widely varying properties. Experiments show that the MXene obtained under the experimental conditions of a high temperature environment, high HF concentration and sufficient etching time will endow more defects and thinner dimensions. Correspondingly, MXene’s electrochemical performance will also be improved [65,66]. More precisely, the variation of etching conditions still depends on the chemical properties of the precursor MAX. When etched only with pure HF, it is still a multi-layer material at the nanoscale, which is similar to an organ shape (Figure 2b). After extensive research, the use of organic base solutions (urea, dimethyl sulfoxide, n-butylamine) as intercalating agents is an effective way to obtain 2D MXene nanosheets [67].

Due to the danger of HF, other etchants must be found. In 2014, Gogotsi et al. used lithium fluoride (LiF) and hydrochloric acid (HCl) as etchants to prepare Ti_3_C_2_T_x_ MXene for the first time, and showed good electrochemical performance when using it as the electrode material in pseudo-capacitors [68]. This important work sets a precedent for the study of fluoride etchants. Liu et al. used LiF, NaF, NH_4_F and KF·2H_2_O as etchants to etch Ti_3_AlC_2_ and Ti_2_AlC with HCl, and studied the corresponding surface structures of MXenes etched by different etchants [69]. The results show that after the precursor is etched, Li^+^, Na^+^ and K^+^ cations will remain on the surface of Ti_3_C_2_ and Ti_2_C, which forms a MXene with new features. The residual mode and amount of these cations and how to use these new MXenes reasonably are the topics that should be studied in the next step. Liu et al. obtained 2D V_2_C MXene by etching V_2_AlC with NaF and HCl at 90 °C, and it showed excellent electrochemical performance in LIBs. This also demonstrates the availability of fluoride salts as etchants [70]. At present, among all the methods for synthesizing MXene, the LiF/HCl system is still the most used. Compared with the HF system, the LiF/HCl system can not only improve the safety of the experiment but also enlarge the interlayer spacing of MXene (lattice parameter of 40 Å), which is of great significance for the modification of MXenes.

The above methods are all carried out in a fluorine-containing solution. In order to seek a more environmentally friendly way, some fluorine-free etching methods have been developed. Green et al. invented the electrochemical etching method, which is the first report to achieve fluorine-free etching. Ti_2_CT_x_ MXene was successfully prepared by electrochemical etching in a Ti_2_AlC-HCl-platinum (Pt) electrolytic cell. Unfortunately, a too long etching time or a too high cathode potential can easily cause both Al and Ti to be etched away, so this method is not suitable for the preparation of high-purity, high-yield MXene [71]. In contrast, the Ti_3_AlC_2_ two-electrode system designed by Feng et al. achieved a single- and double-layer yield greater than 90% and the supercapacitor showed that its performance was superior to that of the MXene electrode produced by the LiF/HCl system (Figure 2c,d) [72]. Also using MXene as a supercapacitor electrode, Shi et al. designed an iodine (I_2_)-assisted etching method. This method significantly increases the content of oxygen (O) groups. Surprisingly, the environmental stability of the resulting MXene is greatly increased [73]. Thence, the relationship between the stability of MXene and the content of O functional groups is a worthy research direction. In addition, Li et al. synthesized multilayer Ti_3_C_2_T_x_ MXene with 92% purity by an alkali-assisted hydrothermal method. This approach increased the proportion of O functional groups and found TiO_2_ on the surface. However, due to the high NaOH concentration and the experimental temperature of 270 °C, this is not a simple and safe preparation method [40]. Recently, a number of research groups have reported a Lewis acidic molten salt replacement method (Figure 2e,f). The A element in MAX is replaced by ZnCl_2_, KCl-LiCl_2_ or CuCl_2_ in the molten state and the excess molten salt will be stripped from the replaced new MAX phase (Ti_3_ZnC_2_, Ti_2_ZnN, Ti_2_ZnC and V_2_ZnC) due to strong Lewis acidity [74,75,76]. Although this method requires a high temperature, its green form is also worth popularizing. Most importantly, the practical performance of MXene with new features resulting from different surface terminations has made everyone full of longing.

Thus far, Ti_3_C_2_T_x_ MXene is the most studied in the huge family of MXenes and there are many undiscovered new members waiting to be researched. Although many methods for synthesizing MXenes have been developed, they cannot be popularized due to the difficulty and particularity of the methods. As the saying goes, a radish has a pit. Changes in etching conditions mean that MXenes have different properties. Controlling the type and quantity of functional groups is of great significance to the practical application of MXene. For that, combining density functional theory (DFT) calculations with specific experiments is an effective way to guide the synthesis of target MXene.

### 2.2. Superior Properties for LSBs

Since the MXene family is extraordinarily large, their properties vary enormously. Even the Ti_3_C_2_T_x_ MXene, which is the most studied today, can have different properties through the control of the surface terminations. In this section, we only discuss the properties related to LSBs among the many properties of MXenes, while other properties will not be discussed.

In LSBs, one of the most important indicators is the conductivity of the material. The conductivity is directly related to the rate performance of LSBs. There have been numerous reports demonstrating the excellent electrical conductivity of MXene. The conductivity of the single layer Ti_3_C_2_T_x_ MXene was tested to be 6700 S·cm^−1^, which is sufficient to support the kinetics of redox reactions in LSBs [77]. However, the conductivity of MXene prepared by different methods varies. The conductivity of the same Ti_3_C_2_T_x_ MXene film was 5.25 × 10^5^ S·m^−1^ prepared by spin coating and 1.51 × 10^6^ S·m^−1^ by scratch coating [78,79]. Furthermore, for the same MXene, the composition of its surface terminations is a key determinant of conductivity, as confirmed by Vladislav et al. through the synthesis of Nb_2_CT_x_ with different surface terminations [75].

The difference in density between Li_2_S and S_8_ leads to a high degree of volume expansion in LSBs. Therefore, good mechanical properties are also one of the requirements for the electrode material of LSBs. MXene has excellent mechanical strength due to a strong covalent M-X bond. The use of MXene-based electrodes ensures that LSBs do not cause structural collapse during charging and discharging. DFT calculations show that the mechanical strength of M_2_X is greater than M_3_X_2_ and M_4_X_3_ [80]. The mechanical properties of different MXenes need to be studied. Hollow cylinders made up of 5 μm thick Ti_3_C_2_T_x_ paper can support objects 4000 times heavier than themselves, and with 10% polyvinyl alcohol reinforcement this figure can reach 15,000 [81]. This clearly demonstrates the potential of MXene in LSBs.

**Figure 3:**
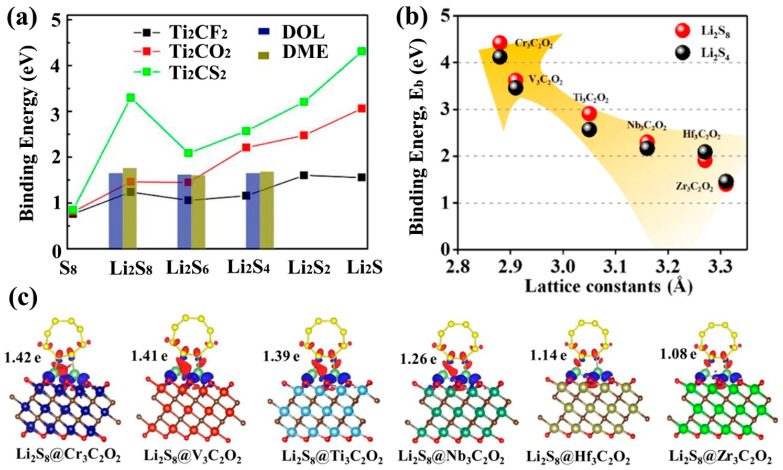
(**a**) Comparison of binding energies between LiPSs and Ti_2_CT_x_ with different surface terminations [85]. Copyright 2018, Elsevier. The binding energies (**b**) and differential charge density (**c**) for Li_2_S_8_ and Li_2_S_4_ as a function of lattice constants of M_3_C_2_O_2_ (M = Zr, V, Ti, Nb, Hf and Cr) MXenes [87]. Copyright 2019, RSC Pub.

In addition to these properties, it is important that the shuttle effect is suppressed by strong interactions with LiPSs. The surface terminations of MXene are the key to the adsorption of LiPSs. The type and number of functional groups and their specific mechanisms to inhibit shuttling of LiPSs are the focus of study. Lu et al. investigated the effect of interaction between Ti_2_C without surface terminations and LiPSs [82]. The DFT calculations revealed that a strong Ti-S bond formed by the S element in LiPSs and the Ti element in Ti_2_C hindered the reversible reaction. The introduction of terminations Ti_2_CF_2_, Ti_2_C(OH)_2_ and Ti_2_CO_2_ enhanced the performance of LSBs by weakening the strong Ti-S bond. Thus, it is sufficient to see the importance of MXene surface terminations. Ti_2_CO_2_ and Ti_3_C_2_O_2_ MXenes with -O terminations exhibit a dual adsorption mechanism in LSBs, Li-O adsorption and Ti-S adsorption. The negatively charged O atom at the end combines with the positively charged Li^+^ in the LiPSs, resulting in a binding energy of around 1–2 eV [83]. Specifically, the presence of Li-O bonds weaken the Li-S interactions in higher-order LiPSs, causing them to convert to lower orders and accelerating the reaction process. The anchoring behavior of O/F-functionalized Ti_2_C MXene on LiPSs was investigated using DFT calculations by Chung et al. [84]. They suggested that neither a single O-functionalized surface nor an F-functionalized surface is as strong as multiple functional group-functionalized MXene for the adsorption of LiPSs. The elimination of surface functional group vacancies is a more effective measure. In addition, Ti_2_CS_2_ MXene with -S terminations exhibits a stronger anchoring ability to LiPSs than O/F surface-terminated MXene (Figure 3a). Theoretical calculations on single-layer Ti_2_C and Ti_2_CS_2_ without adsorbed LiPSs show that Ti_2_CS_2_ retains metallic properties and thus promotes the electrochemical activity during charging and discharging [85]. In addition, titanium nitride MXene (Ti_2_N) is also considered as a promising cathode host material for LSBs. Lin investigated the interaction of O-functionalized and F-functionalized Ti_2_N with LiPSs using first-principle calculations. The results showed that both Ti_2_NO_2_ and Ti_2_NF_2_ have moderate adsorption energies with LiPSs [86]. New developments have also been made in the interaction of members other than Ti-based MXenes with LiPSs. The anchoring behavior of six -O termination MXenes to LiPSs (Cr_3_C_2_O_2_, V_3_C_2_O_2_, Nb_3_C_2_O_2_, Hf_3_C_2_O_2_, Zr_3_C_2_O_2_ and Ti_3_C_2_O_2_) was investigated by Fan et al. [87]. The results show that Cr_3_C_2_O_2_ has the strongest anchoring effect on Li_2_S_4_ and Li_2_S_8_ (Figure 3b,c). It is worth noting that due to the considerable number of MXene family members, exploring the ability of the different surface ends of different MXene species to interact with LiPSs remains an important challenge.

In summary, MXenes possess advantageous properties, such as metallic conductivity, mechanical toughness, structural diversity and adsorption of LiPSs. This not only promotes redox reaction kinetics through high electron mobility, but also suppresses shuttle effects through functionalized MXene terminations. Most importantly, its partially functionalized terminations allow MXene to retain metallic properties that two-dimensional materials such as graphene cannot possess. Thus, MXenes are extremely promising in LSBs.

## 3. MXenes in Li–S Batteries

### 3.1. MXenes as Sulfur Hosts

As mentioned above, MXenes have excellent conductivity, diverse functional terminations and are a reasonable material choice for LSBs cathodes. In this section, we summarize the research progress of MXene-based LSBs sulfur hosts from three aspects: pure MXenes, modified MXenes and MXenes composites.

#### 3.1.1. Pure MXenes as Sulfur Hosts

Layered Ti_3_C_2_ (L-Ti_3_C_2_) was used as a sulfur host for the first time by Zhao et al. [88] Due to the low specific surface area (SSA) of L-Ti_3_C_2_ (7.8 m^2^·g^−1^), the sulfur cathode (S/L-Ti_3_C_2_) had only 57.6% sulfur loading. Nevertheless, the S/L-Ti_3_C_2_ cathode still provided an initial discharge capacity of 1291 mAh·g^−1^ at a current density of 200 mA·g^−1^ and still had 970 mAh·g^−1^ remaining after 100 charge–discharge cycles. Subsequently, after the LiF/HCl method had matured, Nazar et al. synthesized Ti_2_C with an SSA of 67.9 m^2^·g^−1^ and obtained a 70% sulfur loading cathode (70S/Ti_2_C) with a remaining discharge capacity of 723 mAh·g^−1^ at 0.5 °C after 650 cycles [53]. These two studies show that Ti_3_C_2_ and Ti_2_C are full of endless potential in the field of cathodes for LSBs.

**Figure 4:**
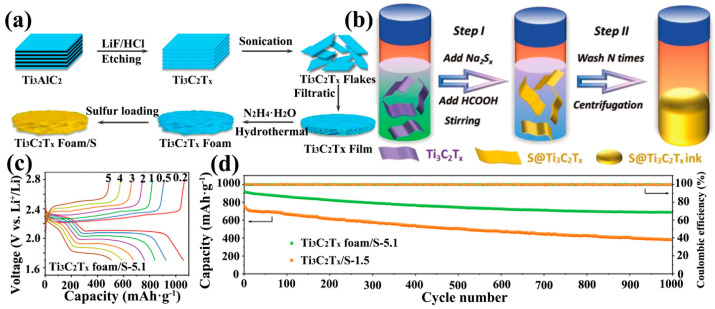
Synthesis of Ti_3_C_2_T_x_ foam/S cathodes (**a**) and their corresponding cell-rate performance (**c**) and long-cycle performance (**d**) [89]. Copyright 2018, RSC Pub. (**b**) Synthesis of S@Ti_3_C_2_T_x_ ink [91]. Copyright 2018, John Wiley and Sons.

**Figure 5:**
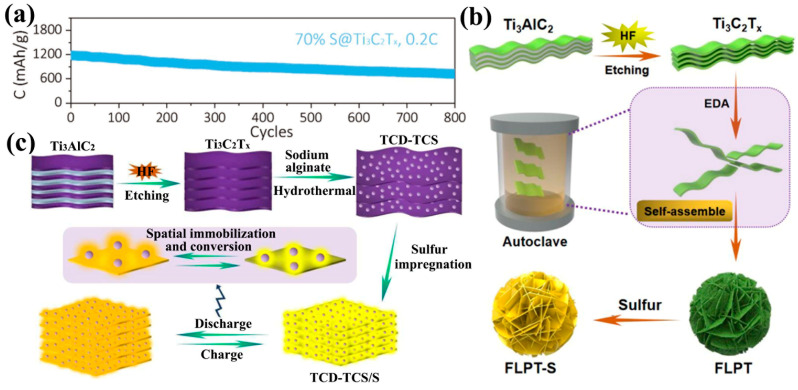
(**a**) Long-cycle performance of S@Ti_3_C_2_T_x_ ink electrode at 0.2C [91]. Copyright 2018, John Wiley and Sons. (**b**) Schematic diagram of the synthesis of FLPT-S cathode [92]. Copyright 2019, American Chemical Society. (**c**) Schematic diagram of the synthesis of TCD-TCS/S cathode [93]. Copyright 2019, American Chemical Society.

Due to its excellent mechanical properties and two-dimensional morphology, Ti_3_C_2_T_x_ can be extracted into thin films and be directly used as a sulfur host substrate for LSBs. Ti_3_C_2_T_x_ flakes are self-stacked into thin films with excellent electrical conductivity, eliminating the need for traditional slurry coating processes, reducing the use of collectors, binders and conductive agents, and reducing costs. Wang et al. soaked freestanding Ti_3_C_2_T_x_ films in hydrazine monohydrate and obtained Ti_3_C_2_T_x_ foams by the hydrothermal method (Figure 4a) [89]. The SSA of the hydrothermally-reacted Ti_3_C_2_T_x_ foam was 182.9 m^2^·g^−1^, which was much larger than that of the Ti_3_C_2_T_x_ film (26.3 m^2^·g^−1^). Thus, the sulfur loading of the Ti_3_C_2_T_x_ foam cathode could reach 5.1 mg·cm^−2^ after compounding with sulfur in carbon disulfide solution (S/CS_2_). Even at high sulfur loading, the Ti_3_C_2_T_x_ foam cathode showed excellent rate capability (Figure 4c) and stable long-cycle capability (Figure 4d). Loading sulfur nanoparticles on Ti_3_C_2_T_x_ thin films by physical vapor deposition (PVD) is also an effective strategy. The Ti_3_C_2_T_x_/S paper prepared by this method possesses excellent mechanical properties and is one of the perfect candidates for flexible LSB cathodes [90]. In addition, the direct preparation of Ti_3_C_2_T_x_/S cathodes is also a reasonable strategy [91]. Sulfur was directly compounded in the process of MXene film formation to generate a Ti_3_C_2_T_x_/S cathode in one step. In detail, the Ti_3_C_2_T_x_ nanosheet solution was mixed directly with sodium polysulfide (Na_2_S_4_) solution and then formic acid solution (HCOOH) was added dropwise to make the sulfur nanoparticles adhere uniformly to Ti_3_C_2_T_x_, which eventually formed the S@Ti_3_C_2_T_x_ cathode directly (Figure 4b). S@Ti_3_C_2_T_x_ with 70% sulfur loading showed only 0.048% capacity decay per cycle in 800 cycles at 0.2 C rate (Figure 5a).

Enhancing the SSA and electron transfer rate of MXene by changing its morphology can be an effective method to enhance the electrochemical performance of LSBs. Wang and co-workers prepared flower-like porous Ti_3_C_2_T_x_ as a sulfur host material (FLPT-S) (Figure 5b) [92]. An ultrahigh volumetric capacity of 2009 mAh·cm^−3^ was obtained for the FLPT-S cathode without adding any carbon conductive agent. Subsequently, they also reported that the Ti_3_C_2_T_x_ nanodots were uniformly dispersed on Ti_3_C_2_T_x_ nanosheets (TCD-TCS) to enrich the polar sites of the cathode, and the TCD-TCS/S cathode with 13.8 mg·cm^−2^ surface density exhibited an ultrahigh volumetric capacity of 1957 mAh·cm^−3^ (Figure 5c) [93].

#### 3.1.2. Modified MXenes as Sulfur Hosts

Chemical modification of the sulfur host material is thought to be effective in inhibiting the shuttling of LiPSs. The doping of elements such as N and O into the surface of nonpolar carbon materials can greatly strengthen their interactions with sulfur [29,94]. This also applies to polar MXene. The synergistic adsorption of LiPSs by heteroatoms and MXene surface terminations is a more effective way to enhance the electrochemical performance of LSBs.

**Figure 6:**
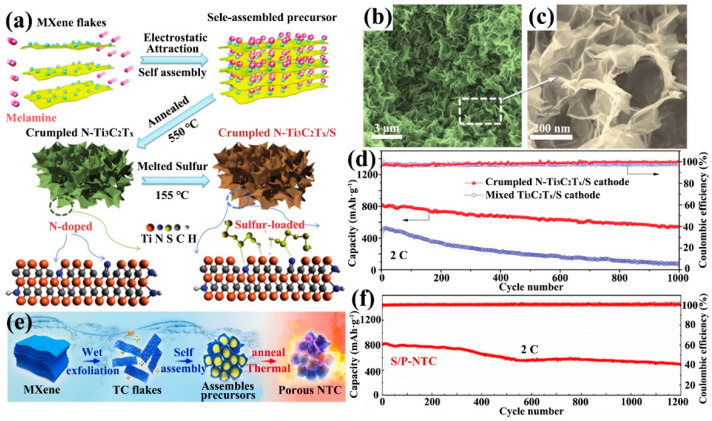
Preparation of crumpled N-Ti_3_C_2_T_x_/S cathode (**a**), SEM images (**b**,**c**) and its 1000 cycles test at 2C (**d**) [95]. Copyright 2018, John Wiley and Sons. Synthesis diagram of P-NTC (**e**) and 1200 cycles of S/P-NTC test at 2C (**f**) [96]. Copyright 2020, Elsevier.

The N element is one of the most studied elements among all doped systems. The chemisorption of pyridinic N to LiPSs should not be underestimated. Wang et al. synthesized crumpled nitrogen-doped Ti_3_C_2_T_x_ nanosheets (N-Ti_3_C_2_T_x_) by a one-step thermal treatment method using melamine as the nitrogen source (Figure 6a) [95]. The crumpled structure allowed for better physical encapsulation of the sulfur nanoparticles (Figure 6b,c). The decomposition of melamine during annealing increased the SSA of Ti_3_C_2_T_x_ nanosheets to 385 m^2^·g^−1^, which was sufficient to give a higher sulfur loading to the cathode host material (N-Ti_3_C_2_T_x_/S). Pyridinic N accounted for 34.18% of all N content, enhancing the interaction of polar Ti_3_C_2_T_x_ with LiPSs. The N-Ti_3_C_2_T_x_/S cathode still had a specific capacity of 610 mAh·g^−1^ after 1000 cycles at a high rate of 2 C (Figure 6d). For a high sulfur loading cathode of 5.1 mg·cm^2^, it still had a good performance. Moreover, for N-Ti_3_C_2_T_x_, Sun et al. obtained porous N-doped Ti_3_C_2_ (P-NTC) using the melamine formaldehyde (MF) template method (Figure 6e) [96]. MF microspheres disappeared after thermal annealing and the sulfur nanoparticles occupied the MF microspheres. So, they were successfully encapsulated in the P-NTC to form a sulfur host cathode (S/P-NTC). This cathode achieved a capacity decay rate of 0.033% at 2 C (1200 cycles) (Figure 6f). The electrochemical properties exhibited by the above two examples are comparable. Meanwhile, this implies a high-quality potential for heteroatom-doped MXene.

**Figure 7:**
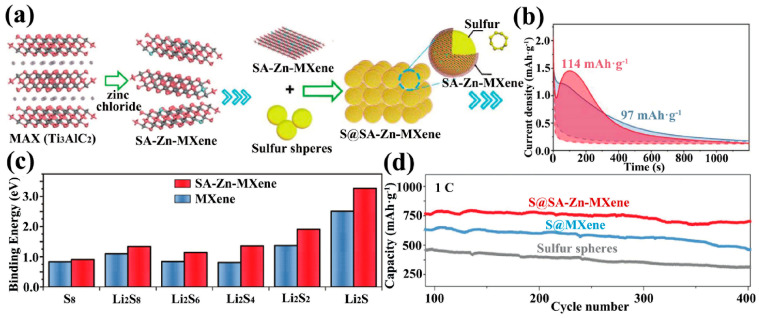
(**a**) Synthesis of SA-Zn-MXene@S cathode. (**b**) Potentiostatic nucleation profiles of Li_2_S_2_/Li_2_S on SA-Zn-MXene and MXene at 2.05 V. (**c**) Binding energies between LiPSs and SA-Zn-MXene layers and between LiPSs and MXene layers. (**d**) A total of 400 long-cycle tests with different sulfur cathodes at 1 C [99]. Copyright 2018, John Wiley and Sons.

In addition to non-metal elements, metal elements have been used as catalysts to promote the kinetics of redox reactions. For example, nickel (Ni) atoms were introduced into the graphene skeleton as electrocatalysts to accelerate the liquid–solid phase transition among polysulfides [97]. Iron (Fe) atoms were introduced into the N-doped graphene skeleton to form Fe-N bonds and promote the deposition of solid-state Li_2_S_2_ and Li_2_S [98]. In the MXene skeleton, the doping of metal atoms could be achieved using the Lewis acidic molten salt replacement method mentioned in the previous section on the synthesis strategy. Molten zinc chloride (ZnCl_2_) displaced the Al atoms in Ti_3_AlC_2_ to form single atom zinc implanted MXene (SA-Zn-MXene) (Figure 7a) [99]. Attributed to the electronegativity of Zn, the binding energy of SA-Zn-Mxene to all LiPSs was higher than that of pure Ti_3_C_2_T_x_ to all LiPSs (Figure 7c). In particular, the potentiostatic nucleation profile analyses of SA-Zn-MXene and MXene showed that MXene with Zn atom doping (red region in Figure 7b) possessed higher Li_2_S precipitation capacity than pure MXene (blue region in Figure 7b), which revealed that SA-Zn-MXene accelerated the nucleation process of solid Li_2_S_2_/Li_2_S. The electrode with SA-Zn-MXene as the sulfur host still had a specific capacity of 706 mAh·g^−1^ at 1 C for 400 cycles, which was much better than the pure MXene electrode (Figure 7d).

All the above examples demonstrate the adsorption as well as the catalytic conversion ability of heteroatom-modified MXene on LiPSs. Although different functional groups have been available on the surface of MXenes to adsorb LiPSs, the study of other heteroatom-modified MXenes cannot be ignored. Both theoretical studies and experimental demonstration of the application of modified MXene in LSBs are still lacking.

#### 3.1.3. MXene-Based Composites as Sulfur Hosts

Despite the strong chemisorption of MXene terminations to LiPSs, it is not negligible that van der Waals interactions cause the monolayer MXene sheets to restack, leading to a significant loss of their SSA as well as a decrease in termination utilization. Therefore, compounding of MXene with other materials is needed to reduce its tendency for restacking. Among many materials, the conductive carbon material has the advantage of a large SSA and strong mechanical stability that can load more sulfur as well as ease the expansion of the electrode, even though its interaction with LiPSs is limited. Moreover, the insertion of the conductive carbon material into the MXene interlayer channel effectively prevents its restacking.

**Figure 8:**
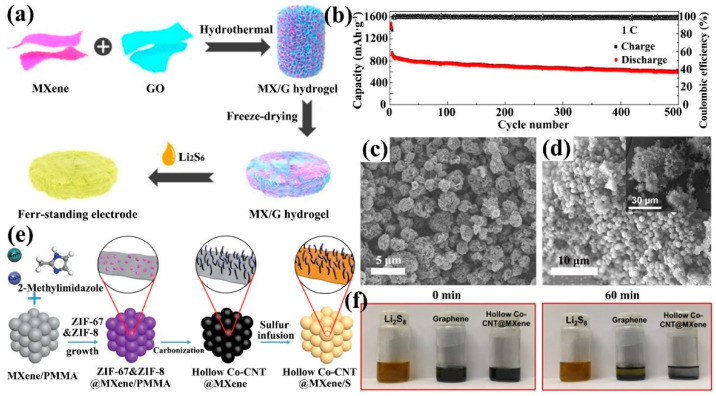
Preparation of MX/G aerogel sulfur cathodes (**a**) and their testing at 1 C for 500 cycles (**b**) [101]. Copyright 2019, Royal Society of Chemistry. (**c**) SEM image of N-Ti_3_C_2_@CNT microspheres [102]. Copyright 2019, Springer. SEM image of hollow Co-CNT@MXene microspheres (**d**) and the fabrication process of their supported sulfur cathodes (**e**). The adsorption test of LiPSs by hollow Co-CNT@MXene microspheres, after 60 min the LiPSs solution of hollow Co-CNT@MXene microspheres became obviously clearer than the LiPSs solution of graphene (**f**) [103]. Copyright 2020, Elsevier.

Graphene, as a representative of carbon-based 2D materials, is an excellent choice for composite materials. The Ti_3_C_2_T_x_ solution after HF etching was washed by graphene oxide (GO), and then Ti3C2Tx and reduced graphene oxide complex (Ti_3_C_2_T_x_/rGO) were obtained by reduction with hydrazine hydrate and sonication. The capacity of the sulfur cathode obtained after liquid-phase carbon disulfide (CS_2_) dissolution of sulfur at 0.5 C for 300 cycles was only reduced to 265.8 mAh·g^−1^, which was much better than that for the restacked Ti_3_C_2_T_x_ cathode [100]. Secondly, graphene can be utilized as 2D nanosheets. By hydrothermal reaction, GO solution can be reduced to rGO aerogel. rGO aerogel with a 3D porous structure was a good sulfur host. It was difficult for MXene to form a 3D structure alone. With the advantage of the 3D rGO aerogel, Ti_3_C_2_T_x_ was compounded with GO to make Ti_3_C_2_T_x_ nanosheets uniformly stacked on rGO to form MXene/rGO composite aerogel (Figure 8a). The cell with this as the sulfur cathode decayed only 0.07% per cycle on average over 500 cycles of 1 C (Figure 8b) [101].

**Figure 9:**
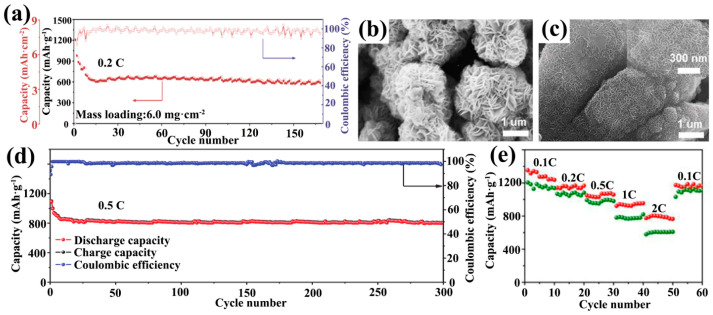
Cycling performance of hollow Co-CNT@MXene/S highly loaded cathode at 0.2 C (**a**) [103]. Copyright 2020, Elsevier. SEM images of 2H MoS_2_ nanoflowers (**b**) and MXene/2H MoS_2_ nanoflowers (**c**). Cycling performance of MXene/1T-2H MoS_2_-C-S electrode at 0.5 C (**d**) [105]. Copyright 2018, John Wiley and Sons. Rate performance of MXene@TiO_2_/S electrodes (**e**) [106]. Copyright 2018, RSC Pub.

Futhermore, carbon nanotube (CNT) additives are highly conductive carbon materials that prevent MXene from restacking. Wang et al. synthesized microspherical MXene/CNT host material by spray drying and one-step pyrolysis [102]. CNTs were uniformly grown on the surface of MXene during pyrolysis and interspersed in the MXene nanosheets. To enhance the chemisorption of the material, N atoms were also doped in the material using melamine as the nitrogen source to form N-doped MXene and CNTs microspheres (N-Ti_3_C_2_@CNT). The microspheres were internally connected by N-Ti_3_C_2_ and N-CNT to form a 3D porous conductive structure, which increased the electron transfer rate and SSA. LiPSs was confined within N-Ti_3_C_2_@CNT microspheres during the redox reaction, and their sulfur electrodes exhibited an initial discharge specific capacity of 1399.2 mAh·g^−1^ at 0.1 C. Also using MXene as a scaffold for the microsphere structure (Figure 8c,d), Zhao and co-works prepared CNT@MXene precursors by growing zeolitic imidazolate frameworks (ZIF) on N-MXene material and the precursors were successfully prepared as hollow cobalt-doped CNTs and Ti_3_C_2_T_x_ composites (hollow Co-CNT@MXene) after pyrolysis (Figure 8e). The Co nanoparticles encapsulated within the CNT synergistically adsorbed LiPSs with the Ti_3_C_2_T_x_ terminations, which made the Li_2_S_8_ solution clear quickly (Figure 8f). Meanwhile, the Co-CNT@MXene/S shows a capacity retention of 85.8% after 170 cycles at 0.2 C (Figure 9a) [103]. The above two examples of CNT@MXene were not enough to guarantee the success rate of CNT-limited MXene restacking, and the advantage of its better performance might be derived from the doping of N or Co atoms and its unique microsphere structure. Specifically, the surface curvature of CNT was high enough that it interspersed with the MXene nanosheets and effectively limited the amount of MXene restacking. Carbon fiber (CF) could overcome this disadvantage. A 10μm diameter CF compounded with MXene could better limit its stacking tendency. In addition, CF has high fiber ductility and is able to maintain the integrity of the electrode structure. In terms of performance, the cathode with 4 mg·cm^−2^ sulfur loading (Ti_3_C_2_@CF-S) shows a first discharge specific capacity of 1175.2 mAh·g^−1^ at 0.5 C, which was better than that of CNT [104].

Recently, the compounding of MXene with transition metal sulfides and oxides has received increasing attention. Sulfides provide corresponding chemisorption sites for LiPSs, which has long been studied in graphene-based sulfur cathodes [26,107], but has just begun in MXene-based sulfur cathodes. Guo et al. investigated the performance of MXene and molybdenum disulfide (MoS_2_) composite sulfur cathode (MXene/1T-2H MoS_2_-C-S) in a soft-pack battery [105]. The prepared MoS_2_ was nanoflower-like, which was uniformly attached to the surface of MXene and rich in defects (Figure 9b,c). The active sites generated by these defects were the main reason for the inhibition of the diffusion of LiPSs. The soft-pack battery with MXene/1T-2H MoS_2_-C-S cathode shows an initial discharge capacity of 1014.1 mAh·g^−1^ at 0.5 C and maintains 799.3 mAh·g^−1^ after 300 cycles, showing excellent cycling stability (Figure 9d). In the oxide direction, there are many studies on titanium dioxide (TiO_2_). TiO_2_ has shown inhibition of LiPSs in several system studies [108,109]. It is well known that Ti_3_C_2_T_x_ MXene is easily oxidized to TiO_2_ in air. Thus, Wu et al. successfully constructed irregular MXene/TiO_2_ heterostructures by in situ oxidation of Ti_3_C_2_ nanosheets. After loading sulfur by a simple melt-diffusion method (MXene/TiO_2_/S), sulfur was uniformly attached to the MXene/TiO_2_ heterostructure. The MXene/TiO_2_/S cathode maintained a capacity of 774.7 mAh·g^−1^ at 2 C and recovered 1174.8 mAh·g^−1^ after returning to 0.1 C (Figure 9e), demonstrating excellent multiplicity performance [106]. Du et al. encapsulated sulfur in TiO_2_ hollow spheres and embedded them into Ti_2_C interlayers, this electrode maintained a capacity of 227.3 mAh·g^−1^ after 200 cycles at 5C [110]. Wen et al. used NH_4_BF_4_ /HCl etching to grow AlF_3_ nanoparticles on MXene and then introduced Ni(OH)_2_ nanosheets as a physical baffle to hinder LiPSs; this cathode exhibited an extremely low capacity decay rate in 1000 cycles of 1C (0.048% per cycle) [111].

Covalent organic frameworks (COFs), a new class of two-dimensional materials with high specific surface area, have been extensively investigated in LSBs in recent years. A novel two-dimensional sulfur host material (CTF/TNS) was constructed by Meng et al. using the ordered porous structural characteristics of a COF and compounding it with MXene. This material was able to maintain 94% capacity after 100 cycles at a high sulfur loading of 5.6 mg·cm^−2^. This demonstrates that the CTF/TNS heterostructure plays an exceptionally critical role in promoting Li^+^ diffusion and adsorption of LiPSs [112].

All the above studies showed that building sulfur hosts around MXene was an effective strategy to boost the electrochemical performance of LSBs. The superior conductivity of MXene enabled the cells to exhibit high-rate performance. Furthermore, its effective terminations can effectively capture LiPSs. The disadvantage of the easy stacking of MXene can also be solved to a great extent by introducing carbon materials or metal compounds. However, modified MXene has received little attention. Even Ti-based MXene, which has been widely studied nowadays, has rarely been studied in doping. The electrical conductivity of modified MXene with different atoms and the adsorption ability of the surface terminations have not yet been clearly explained. Theory and experiments are urgently required to demonstrate their potential.

### 3.2. MXenes as Interlayers

The separator, a key component in preventing internal short circuit and facilitating ion diffusion, also affects the performance of the cell. Most of the separators used in laboratory assembly of LSBs are polypropylene separators (PP), but their poor permeation effect leads to slow kinetics of LiPSs. Introducing an interlayer between the cathode and the separator for trapping LiPSs is an effective strategy to enhance the performance. Independent interlayer films, double-layer cathodes and functional separators are examples of interlayer applications. Some progress has been made in using MXene-based materials as the interlayer materials of LSBs.

**Figure 10:**
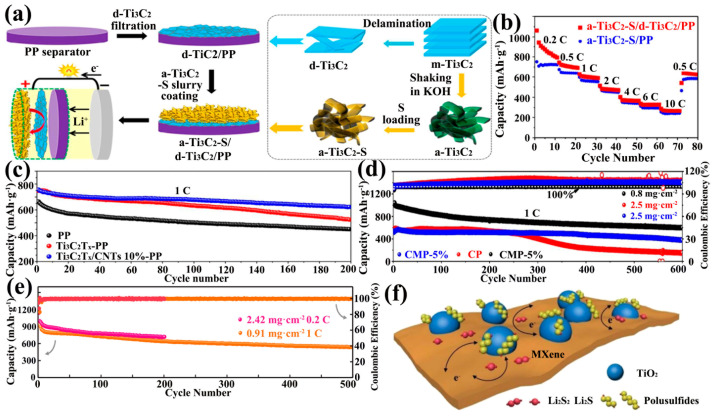
Preparation process of a Ti_3_C_2_-S/d-Ti_3_C_2_/PP electrode for LSBs (**a**) and its rate performance (**b**) [114]. Copyright 2018, American Chemical Society. Long-cycle performance of LSBs of Ti_3_C_2_T_x_/CNTS composite interlayer by different workers (**c**–**e**) [115,116,117]. Copyright 2020, Elsevier. Copyright 2019, Elsevier. Copyright 2021, John Wiley and Sons. (**f**) Schematic illustration of the interaction between the TiO_2_-Ti_3_C_2_T_x_ heterostructure and LiPSs [118]. Copyright 2019, John Wiley and Sons.

Pure MXene can be used as an interlayer due to the high conductivity and high adsorption terminations of MXene. The dispersion of Ti_3_C_2_T_x_ and ethanol was attached to the PP separator by vacuum filtration to obtain an interlayer with a loading of 0.1 mg·cm^−2^. The initial discharge capacity of LSBs with sulfur–carbon (S/C) material, with a sulfur loading of 1.2 mg·cm^−2^, as the cathode reached 1246.3 mAh·g^−1^ at 0.2 C [113]. Subsequently, an all-MXene-based integrated electrode (a-Ti_3_C_2_-S/dTi_3_C_2_/PP) was designed by Dong et al. (Figure 10a). MXene was used as both the sulfur host and the interlayer and no Al collector was used for the cathode. The functional separator was also prepared using the vacuum filtration method with a loading of 0.4 mg·cm^−2^. The all-MXene-based integrated electrode cell showed an excellent rate capacity of 288 mAh·g^−1^ at 10 C (Figure 10b) [114].

Pure MXene shows limited adsorption ability on LiPSs. Its composite with nonpolar carbon showed better performance in the sulfur host cathode. For the interlayer, Ti_3_C_2_T_x_ and GO were vacuum filtered to obtain Ti_3_C_2_T_x_/GO free-standing membranes. The addition of GO provided physical restriction for LiPSs and prevented the movement of Li^+^ to the anode. Combining with the strong adsorption of MXene terminations on LiPSs, the Ti_3_C_2_T_x_/GO system restricted the diffusion of LiPSs from both physical and chemical aspects. Cells with a Ti_3_C_2_T_x_/GO interlayer show an ultra-high initial discharge capacity of 1621.5 mAh·g^−1^ at 0.1 C [119]. CNTs have also been applied in the interlayer of LSBs as one of the carbon materials capable of avoiding the restacking of Ti_3_C_2_T_x_ nanosheets. Moreover, CNTs had the ability to enhance the electrical conductivity in the cross-sectional direction of the MXene layer. Li et al. prepared Ti_3_C_2_T_x_/CNT functional separators with a mass loading of 0.016 mg·cm^−2^. The Ti_3_C_2_T_x_/CNT functional separators doped with only 10% CNT significantly outperformed the undoped CNT separators in the 1 C cycle performance (Figure 10c) [115]. In other work, the loading of Ti_3_C_2_T_x_/CNTs was boosted to 0.16 mg·cm^−2^ but the MXene content was 5% of the total mass. Again, this work showed better performance in 1 C cycling performance (Figure 10d) [116]. Recently, Ti_3_C_2_T_x_/CNT modified separators with about 10% CNT were also designed by Yang et al. for inhibiting LiPSs and accelerating Li^+^ diffusion. The difference from the two previous studies was that the Ti_3_C_2_T_x_ flakes were treated with different concentrations of copper sulfate solution (CuSO_4_) to obtain materials with different pore densities and sizes, and the loading was further increased to 0.5 mg·cm^−2^. The PM (0.4 M)-CNT interlayer (0.4 M = Cu^+^ concentration) did not show better long-cycle performance than the above work at 1 C (Figure 10e) [117].

**Figure 11:**
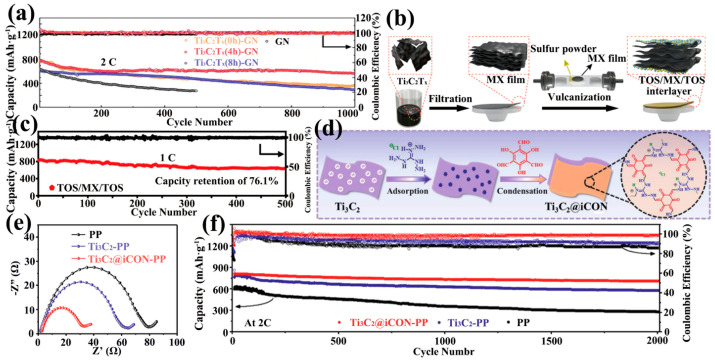
Long cycling of LSBs with the TiO_2_-Ti_3_C_2_T_x_ heterostructure as the interlayer at 2 C (**a**) [118]. Copyright 2019, John Wiley and Sons. Schematic illustration of the synthesis process of the TOS/MX/TOS sandwich structure (**b**). The 500-cycle performance of LSBs with TOS/MX/TOS sandwich structure as the interlayer at 1 C shows a capacity retention of 76.1% (**c**) [120]. Copyright 2022, Elsevier. Schematic diagram of the synthesis of Ti_3_C_2_@iCON heterostructures (**d**). EIS performance of LSBs with Ti_3_C_2_@iCON-PP separator (**e**) and its long-cycle performance at 2 C (**f**) [121]. Copyright 2021, John Wiley and Sons.

TiO_2_/Ti_3_C_2_T_x_ composites have been widely noticed. By controlled oxidation of Ti_3_C_2_T_x_, the terminal F was replaced by O to form the TiO_2_-MXene heterostructure (Figure 10f). TiO_2_ maintained the 2D structure in the heterostructure to provide a large SSA and catalyzed the conversion of LiPSs. The TiO_2_-MXene heterostructure was mixed with graphene to form the Ti_3_C_2_T_x_(0,2,4,6,8h)-GN interlayer (0, 2, 4, 6, 8 h was the oxidation time). LSBs with this as an interlayer exhibit a high capacity of 800 mAh·g^−1^ at 2 C and a capacity decay rate of only 0.028% over 1000 cycles (Figure 11a) [118]. In addition, besides controlled oxidation, sulfation of MXene has been investigated. The sandwich structured TiS_2_-TiO_2_/Mxene interlayer (TOS/MX/TOS) was obtained by heating MXene membranes and sulfur powder by inert gas in a tube furnace (Figure 11b). TiO_2_ acts as an adsorbent for LiPSs. TiS_2_ acts as a catalyst for the redox reaction and MXene provides high electrical conductivity and high SSA. LSBs with the synergistic effect of the three were up to 76.1% capacity retention at 1 C for 500 cycles (Figure 11c) [120].

In recent years, COFs based on guanidinium salts have attracted attention due to their strong covalent bonds and abundant pore channels [108,109]. It has been proposed that guanidinium salts could adsorb polysulfides due to electrostatic interactions [122]. Li et al. treated ionic covalent organic nanosheets (iCON) based on guanidinium salts as composites to avoid Ti_3_C_2_ restacking and trapping LiPSs (Ti_3_C_2_@iCON) (Figure 11d). Ti_3_C_2_@iCON was uniformly attached to the PP separator with a loading of 0.1 mg·cm^−2^ by the vacuum filtration method. The LSBs with CNT/S as the cathode and Ti_3_C_2_@iCON-PP as the separator exhibited lower charge transfer resistance (Figure 11e) and a capacity decay rate of 0.006% at 2000 cycles at a large rate current of 2 C, with the capacity only decaying from 810 mAh·g^−1^ to 706 mAh·g^−1^ (Figure 11f) [121]. This study demonstrates that the development of ultra-long-life LSBs can be realized. Futhermore, polymers can also be used as intercalation materials between MXene sheets to facilitate Li^+^ transport, and the MXene/Nafion-modified separator designed by Wang et al. was able to provide 794 mAh·g^−1^ capacity at 3 C [123].

All of the above studies have shown that MXene materials can significantly improve the electrochemical performance of LSBs whether they are used for sulfur hosts or interlayers (Table 1). Unfortunately, the MXene-based materials do not have much advantage over the performance exhibited by carbon-based materials for sulfur hosts and interlayers. Therefore, it is urgent to explore other MXene members that inhibit the shuttle effect more significantly and catalyze the conversion of LiPSs more efficiently.

## 4. Summary and Future Perspectives

MXenes have evolved rapidly in recent years and several synthetic methods have been developed for etching MAX precursors. The etch-exfoliated MXenes show metal-level conductivity and larger SSA, which endows MXene with multi-industry applications. Unlike 2D carbon materials, 2D MXene contains abundant terminations, e.g., -O, -F, -OH, etc. These functional groups have different advantages in different fields. In LSBs, surface terminations of MXene show a strong trapping ability for LiPSs, which is significant in suppressing the shuttle effect. When MXene is used as a cathode host for LSBs, its large SSA can increase the load of sulfur. On the one hand, its metal conductivity improves the utilization of sulfur, on the other hand, it realizes high-rate LSBs by providing a high-speed electronic transmission network. When MXene is used as an intermediate layer, it shows the effect of inhibiting LiPSs migration, the terminal functional groups block LiPSs within the cathode region by strong chemical bonding, which can suppress the notorious shuttle effect. Chemically modified MXene or MXene composites can even catalyze electrochemical kinetics. In view of the above properties, MXene-based LSBs have made good progress. Nevertheless, they still face serious challenges to commercialization.

1. Various preparation methods have been developed for the most studied Ti-based MXene so far, including HF etching, fluorine salt etching, electrochemical etching, molten salt replacement, etc. The properties of MXene, such as conductivity, terminal adsorption and structural diversity can be achieved by changing the preparation route. Among the LSBs, only HF and LiF/HCl have been widely used. The remaining etching methods lack profound studies on the modification of MXene properties, especially for MXene surface termination properties, which have a significant impact on the inhibition of the LSBs shuttle effect.
2. After exfoliation, the monolayer MXene is easy to oxidize due to the exposure of surface metal atoms and will also self-restack due to van der Waals interaction forces. Whether it is the cathode or the interlayer of LSBs, the preparation process is indispensable to contact with air. The restacking of MXene results in a smaller SSA, which leads to the agglomeration of the active material sulfur loaded on the MXene sheet layer and the reduced sulfur utilization will directly lead to the reduction of the battery cycle life.
3. The surface terminations of MXene, -OH, -F, -O and -Cl, have been shown to have the ability to adsorb LiPSs as well as having catalytic activity. However, the principles of their specific adsorption mechanisms are still under researched. Advanced techniques such as theoretical calculations, in situ characterization and COMSOL simulations are effective ways to solve these problems. Moreover, the precise control of the functional groups during the synthesis of MXene is difficult. How to enhance the adsorption of controllable terminations on LiPSs, without losing the high conductivity of MXene is a more severe challenge.
4. Modified MXene is underused in LSBs. Modified carbon-based materials have been shown to be effective in enhancing the electrochemical properties of LSBs. Heteroatom modification makes the electrical conductivity, mechanical properties and easy oxidation of MXene change to a certain extent. It interacts with LiPSs in a different way. It is necessary to explore the mechanism of modified MXene in the sulfur host of LSBs as well as in the interlayer.
5. The number of precursor MAX phases is up to more than 100, while only thirty kinds of MXene have been successfully etched, and there are even fewer MXene that can be exfoliated into monolayers; only Ti-based MXene has been widely studied in LSBs. Thus, our current knowledge of the MXene family is only limited to the surface, and there is still a very broad research space to be developed.

In conclusion, accelerating the application of other Ti-based Mxene preparation methods in LSBs, overcoming the oxidation as well as the restacking problems of MXene, advancing the development of modified MXene and expanding new MXene members are challenges that we should overcome. Therefore, the application of the MXene family in LSBs is still in the preliminary stage, which is an extremely promising direction.

Finally, to realize the commercial application of high-energy-density LSBs, it is not enough to limit the performance study to conventional buckle batteries in the laboratory. In today’s lithium-ion battery market, soft-pack batteries are known for their high market share, and soft-pack batteries can maximize the battery energy density. Consequently, it is necessary to study the performance of soft-pack LSBs. Moreover, cost is also one of the most important factors for the commercialization of LSBs. Accurate and detailed cost projections for LSBs are necessary. Sgroi et al. previously conducted an extremely detailed analytical study on the cost of each module component of direct methanol fuel cells (DMFC) in 2016, and this study provided a detailed cost report for the large-scale production of DMFC, which accelerated its commercialization process [124]. In the field of LSBs, a detailed cell cost analysis report is also urgently needed to enhance their commercialization. It is worth noting that there is an extreme lack of LSB cost analysis studies. Based on this situation, in terms of battery performance, a rational design of the cell structure is needed to improve the cycle life and multiplier performance of LSBs from various aspects, such as sulfur surface density, sulfur content, liquid-sulfur ratio and sulfur utilization. In terms of cost, detailed prediction and evaluation of cathode sulfur loading, sulfur-to-carbon ratio, anode lithium metal quality and electrolyte material costs are needed to better promote the commercialization of LSBs.

## Figures and Tables

**Table 1:** Electrochemical performance of MXene-based cathodes and modified separators in LSBs.

Cathode	Separator	Sulfur Loading (mg·cm^−2^)	Sulfur Content (wt.%)	Initial Capacity (mAh·g^−1^)/Rate	Retain Capacity (mAh·g^−1^)/Cycles/Rate	Rate Capacity (mAh·g^−1^)/Rate	Ref.
70S/d-Ti_2_C	PP	1	70	1200/0.2 C	723/650/0.5 C	660/4 C	[53]
S/L-Ti_3_C_2_	PP	-	57.6	1291/200 mAh·g^−^^1^	970/100/200 mAh·g^−^^1^	620/3200 mA·g^−^^1^	[88]
Ti_3_C_2_T_x_ foam/S-1.5	PP	1.5	-	1226.4/0.2 C	375.8/1000/1 C	711/5 C	[89]
Ti_3_C_2_T_x_/S paper	PP	1.88–2.26	-	1383/0.1 C	923.51/1500/1 C	1075/2 C	[90]
S@Ti_3_C_2_T_x_ ink	PP	-	50	1350/0.1 C	1170/175/2 C	1161/2 C	[91]
crumpled N-Ti_3_C_2_T_x_/S	PP	1.5	-	1144/0.2 C	610/1000/2 C	770/2 C	[95]
S/P-NTC	PP	1.4–1.6	80	1072/0.5 C	360.47/600/5 C	792/3 C	[96]
S@SA-Zn-MXene	PP	1.7	90	1136/0.2 C	706/400/1 C	517/6 C	[99]
Ti_3_C_2_T_x_/RGO	PP	1.5	70.4	1190.2/0.2 C	878.4/300/0.5 C	750/5 C	[100]
MX/G-30	PP	1.57	45	1259/0.1 C	596/500/1 C	977/1 C	[101]
N-Ti_3_C_2_ MXene@CNTs/S	PP	1.5	70	1339.2/0.1 C	775/1000/1 C	640.5/4 C	[102]
Co-CNT@MXene/S	PP	2–2.5	70	1210/0.2 C	401.85/840/1 C	765/1 C	[103]
Ti_3_C_2_@CF-S	PP	4	-	1512.7/0.1 C	459.6/1000/2 C	-	[104]
MXene/1T-2H MoS_2_-C-S	PP	2–4	79.6	1194.7/0.1 C	799.3/300/0.5 C	677.2/2 C	[105]
MXene@TiO_2_/S	PP	1.2	75	1481.5/0.5 C	612.7/500/2 C	774.7/2 C	[106]
S/CB	MXene-PP	1.2	68	1046.9/0.2 C	550/500/0.5 C	743.1/1 C	[113]
a-Ti_3_C_2_-S	d-Ti_3_C_2_/PP	0.7–1	-	1062/0.2 C	632/50/0.5 C	288/10 C	[114]
CNTs/S	Ti_3_C_2_T_x_/GO@PP	3–4	70	1621.5/0.1 C	575.7/200/1 C	640/5 C	[119]
S/CNTs	Ti_3_C_2_T_x_/CNTs 10%-PP	1.2	70	≈1100/0.1 C	640/200/1 C	640/2 C	[115]
S/CNTs	CNTs/MXene-PP	0.8–2.5	70	1415/0.1 C	614/600/1 C	728/2 C	[116]
S/KB	PM (0.4 M)-CNT	0.91	85	1105/0.1 C	535/500/1 C	677.6/2 C	[117]
S/CMK-3	Ti_3_C_2_T_x_ (4 h)-GN	1.2	70	800/2 C	576/1000/2 C	663/2 C	[118]
-	TOS/MX/TOS	-	-	961.7/0.2 C	632.8/500/1 C	804.5/1 C	[120]
CNT/S	Ti_3_C_2_@iCON-PP	1.2	-	1417/0.05 C	706/2000/2 C	687/5 C	[121]

## Data Availability

No new data were created or analyzed in this study. Data sharing is not applicable to this article.

## Abbreviations

LIBs	lithium-ion batteries
LSBs	lithium sulfur batteries
2D	two-dimensional
DFT	density functional theory
SSA	specific surface area
PVD	physical vapor deposition
GO	graphene oxide
rGO	reduced graphene oxide
CNTs	carbon nanotubes
ZIF	zeolitic imidazolate frameworks
CF	Carbon fiber
COFs	Covalent organic frameworks
PP	polypropylene separators
S/C	sulfur-carbon
iCONs	ionic covalent organic nanosheets
DMFC	direct methanol fuel cells
